# Atomistic Insights into the Effect of Functional Groups on the Adsorption of Water by Activated Carbon for Heat Energy Storage

**DOI:** 10.3390/molecules29010011

**Published:** 2023-12-19

**Authors:** Xin-Yue Duan, Zeng-Hui Qian, Yong-Xiao Tuo, Liang Gong, Chuan-Yong Zhu

**Affiliations:** College of New Energy, China University of Petroleum (East China), Qingdao 266580, China; duanxy@upc.edu.cn (X.-Y.D.); z21150070@s.upc.edu.cn (Z.-H.Q.); yxtuo@upc.edu.cn (Y.-X.T.); lgong@upc.edu.cn (L.G.)

**Keywords:** adsorption heat storage, activated carbon, functional groups, GCMC

## Abstract

Adsorption heat storage holds great promise for solar energy applications. The development of new adsorbent materials is currently the research focus in this area. The present work designs several activated carbon models with different functional groups, including -OH, -NH_2_, -COOH, and -SO_3_H, and explores the influence of functional groups’ categories and numbers on the water adsorption capacity of the activated carbon using the GCMC method. The adsorption mechanism between functional groups and water molecules is analyzed using density functional theory. The results show that the functional groups could significantly improve the water adsorption capacity of activated carbon due to the hydrogen bond between functional groups and water molecules. In the scope of this paper, under low pressure, the activated carbon with -SO_3_H exhibits the best adsorption capacity, followed by the activated carbon with -COOH. Under low and medium pressure, increasing the number of -SO_3_H functional groups could increase the water adsorption capacity; however, when the pressure is high, increasing the functional group numbers might decrease the water adsorption capacity. As the temperature increases, the water adsorption capacity of activated carbons decreases, and the activated carbon with -SO_3_H is proven to have excellent application prospects in heat energy storage.

## 1. Introduction

Currently, fossil fuels such as coal, oil, and natural gas are still the dominant energy sources worldwide [1]. The overuse of fossil fuels produces massive amounts of greenhouse gases, which cause climate change. In order to reduce greenhouse gas emissions, almost every country is seeking renewable energies to replace fossil fuels. In recent years, solar energy has attracted significant attention as an inexhaustible source of renewable energy and has benefitted from the rapid development of photovoltaic and photoelectric technologies. However, solar energy is intermittent and discontinuous, which results in low utilization efficiency and energy vacancies in residential areas. Thermal energy storage technologies could balance the energy demand between different times (e.g., daytime and night-time; summer and winter) and different regions, and have been regarded as promising technologies for assisting solar energy applications [2,3]. Thermal energy storage technologies are typically divided into three categories, i.e., latent heat storage, sensible heat storage, and thermochemical heat storage [4]. Thermochemical heat storage includes thermal reaction heat storage and adsorption heat storage. Adsorption heat storage refers to heat storage or heat release by adsorption materials during desorption and adsorption [5], which has many advantages compared with other heat storage technologies, including high heat storage capacity and minor thermal losses. This makes the technology much more attractive for solar thermal energy storage [6].

Developing new adsorption materials and the selection of adsorption and adsorbent pairings are the focuses of adsorption technology. In an adsorption heat storage system, water is the most widely used adsorbate due to its high latent heat. Various porous materials (e.g., zeolite, activated carbon, silica gel, and MOF) are used as adsorption storage materials [7,8,9,10,11,12,13,14,15]. Compared with other adsorbents, activated carbon with high porosity and high thermal stability has many advantages in adsorption heat storage. For example, activated carbon can be produced from waste biomass and municipal sludge, which are plentiful and economical sources of raw materials [16]; furthermore, activated carbon–water adsorption systems may require minimal energy for desorption [17]. For instance, Chairunnisa et al. [18] prepared activated carbon from acorn nutshells and enhanced its water adsorption capacity by air oxidation. They claimed that activated carbon–water pairs showed great potential for use for adsorption heat storage. However, it has been found that under medium and low pressure, activated carbon always shows poor water adsorption capacity since its surface is almost hydrophobic, which significantly limits its applications in heat energy storage [19]. Therefore, developing activated carbon with high water adsorption capacity is key to using an activated carbon–water working pair for heat energy storage.

In the past few years, different research groups have devoted many efforts to investigating the factors that influence activated carbon’s water adsorption capacity. For instance, Qian et al. [20] prepared activated carbons with different pore structures and tested their adsorption capacity for water vapor. They pointed out that the pore structures of activated carbon had a significant influence on the adsorption capacity of water vapor. Activated carbon with a larger pore volume and surface area exhibited a strong water adsorption capacity. Horikawa et al. [21] experimentally investigated the influence of temperature on the water adsorption capacity of activated carbon. Their results show that the water adsorption in micropores was independent of temperature. In the temperature range of 263~298 K, the water adsorption capacity of activated carbon increased as the temperature increased. Fletcher et al. [22] tested the activated carbon’s water adsorption capacity at the temperature of 298 K. They found that the higher the concentration of oxygen-containing function groups on the surface of activated carbon, the higher the activated carbon adsorption under low pressure. In addition, Tangsathitkulchai et al. [23] also investigated the influence of oxygen-containing functional groups on the adsorption performance of activated carbon and proved that functional groups on the activated carbon surface strongly influence the adsorption capacity. Although current experimental studies have shown that the water absorption performance of activated carbon can be significantly affected by the introduction of oxygen-containing functional groups by oxidizing activated carbon, few experiments have been reported to systematically compare the effect of a series of element-containing (nitrogen, sulfur, and oxygen) functional groups on the adsorption of water molecules. In addition, the microscopic mechanism of functional groups affecting the adsorption properties of activated carbon is unclear, which poses a challenge for functionalizing activated carbon to obtain the best adsorbent material. Therefore, it is of great significance to systematically study the specific effects of a series of functional groups added to activated carbon on the adsorption performance of water molecules, and to propose the best modified formula for activated carbon. Molecular simulation is an effective method to explore the microscopic behavior of atoms and molecules. The application of molecular simulation can provide predictive, forward-looking, and unique insights to guide the design of desired materials and to help understand the interaction and adsorption processes of the gas skeleton at a more microscopic level. In recent years, molecular simulation technology has developed rapidly and has been widely used in material evaluation, drug design, and water purification [24,25,26,27,28]. Grand Canonical Monte Carlo (GCMC) is a typical molecular simulation method that can obtain the adsorption configuration at the lowest energy of the whole adsorption system and help researchers realize the process of adsorption from the atomic perspective. It has been successfully used in the simulation of various porous adsorption systems such as zeolite [29,30,31], metal–organic frameworks (MOFs) [32,33,34], and activated carbon [35,36,37].

Therefore, the aim of this study is to employ molecular simulation methods to explore the water adsorption characteristics of carbon materials and the influence of various functional groups on these characteristics. In this study, the random structure model of activated carbon was used to systematically study the effect of functional groups on the water absorption performance of activated carbon. The activated carbon was modified by four functional groups (-OH, -NH_2_, -COOH, and -SO_3_H). All structures were studied intensively using multi-scale simulation methods, including Grand Canonical Monte Carlo (GCMC) and Density Functional Theory (DFT) simulations. Initially, the water adsorption isotherm of activated carbon modified by functional groups was calculated using the GCMC simulation method, the specific effects of the types and quantities of functional groups and temperature on the adsorption performance and the adsorption heat of activated carbon were systematically explored, and the optimal functional group modification scheme was found. Subsequently, DFT was used to analyze the difference in charge density between the functional groups and the adsorbed water molecules, and the internal influencing mechanism of the difference in the water adsorption capacity of activated carbon according to different functional groups was clarified. The following parts of this paper are arranged as follows: In Section 2, the physical models of activated carbon and the numerical details are provided. In Section 3, the influence of the temperature, types, and numbers of functional groups on water adsorption performance and adsorption heat are investigated and discussed. In Section 4, we draw our conclusions. The findings of this study are crucial to elucidate the intrinsic relationship between the water adsorption properties of functionalized activated carbon and the types of functional groups, and they hold significant value in designing promising activated carbon materials for thermal energy storage applications. The purpose of this work is to elucidate the intrinsic relationship between the water adsorption properties of functionalized activated carbon and types of functional groups and to design a promising activated carbon heat storage material.

## 2. Results and Discussion

### 2.1. The Influence of Functional Group Types

**Figure 1:**
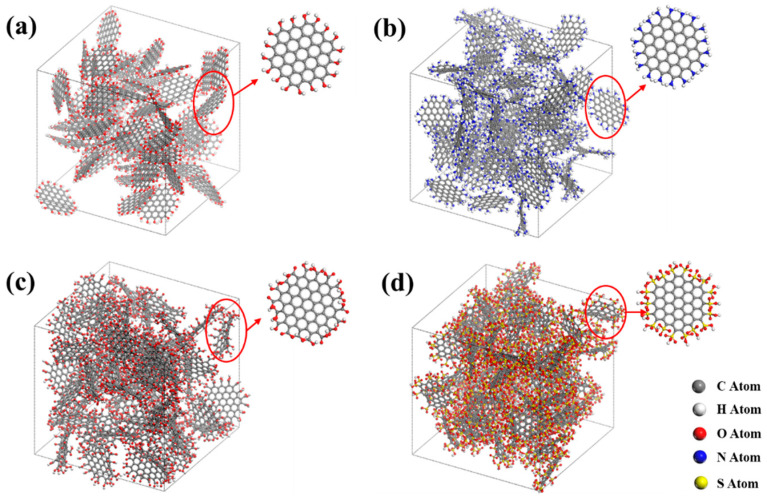
Models of activated carbons with different functional groups: (**a**) -OH; (**b**) -NH_2_; (**c**) -COOH; (**d**) -SO_3_H.

In order to investigate the effect of functional group types on the water adsorption performance of activated carbon, models of activated carbon with four different functional groups, including -OH, -NH_2_, -COOH, and -SO_3_H, were built, as in Figure 1. The functional groups were added to the carbon atoms in 19-ring graphene fragments with unsaturated edge electrons. The size of the simulation domain was 50 Å × 50 Å × 50 Å, containing 58 graphite fragments with functional groups. Porosity is one of the important parameters to characterize the porosity characteristics of porous materials and was calculated by using the probe method in this work. The detailed working principle of the probe method is shown below. When a fixed-diameter probe molecule rolls on the solid skeleton, the surface of the probe in contact with the solid skeleton is the same as that of the van der Waals surface. The volume of the van der Waals surface wrapping is defined as the skeleton volume, and the volume outside the van der Waals surface is defined as the effective pore volume. The detailed structural parameters of different models and the charge of each atom are listed separately in Table 1 and Table 2. It should be noted that the carbon atom in Table 2 is directly connected to the functional group, and the charges of other carbon atoms that are not directly connected to the functional group are set as 0.

**Figure 2:**
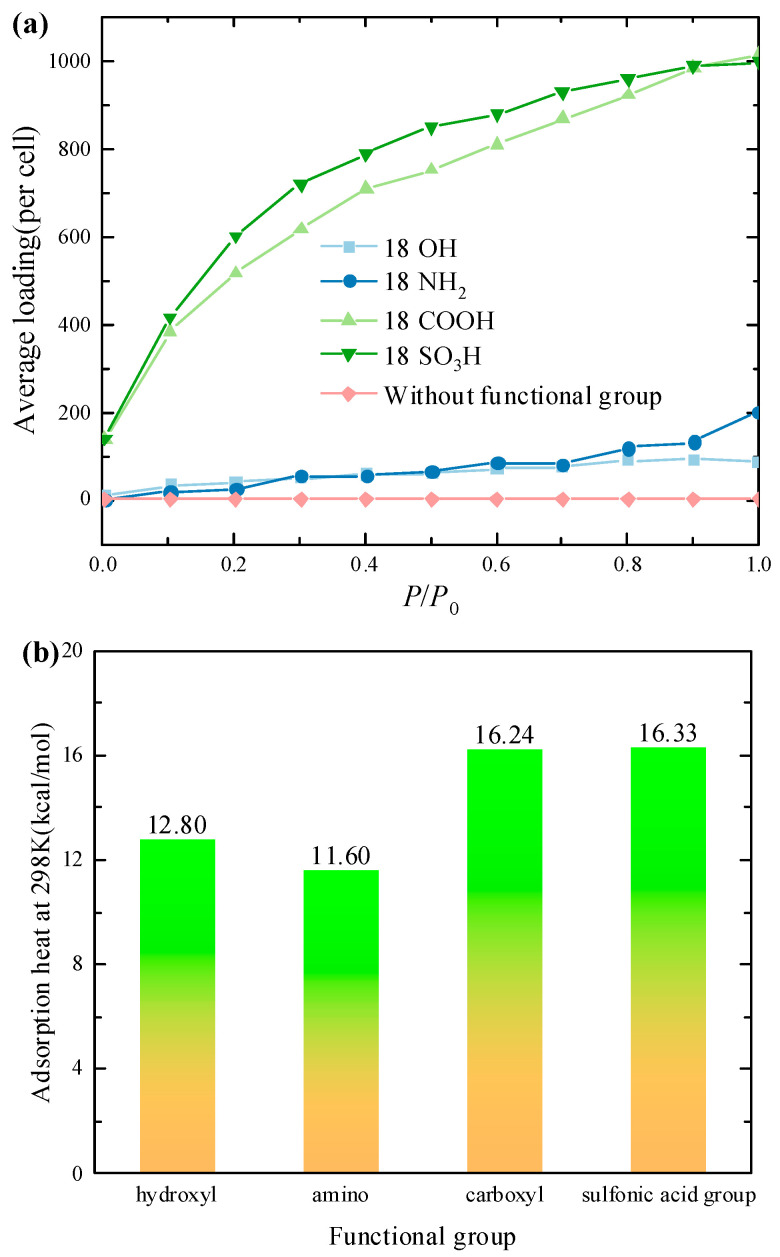
The adsorption isotherm and adsorption heat of activated carbon with different functional groups: (**a**) average loading; (**b**) adsorption heat.

Figure 2a shows the adsorption isotherm of activated carbon at *T* = 298 K with water vapor pressure ranging from 0 kPa to 3.17 kPa. It can be seen from Figure 2 that the pure activated carbon is entirely hydrophobic, and the amount of adsorbed water is almost zero. The activated carbons with -COOH and -SO_3_H have a far better adsorption capacity than activated carbons with -OH and -NH_2_. When the adsorption reaches saturation, the adsorption capacities of activated carbons with -COOH and -SO_3_H are 1014 and 996 per cell, respectively, which are much higher than the adsorption capacities of activated carbons with -OH and -NH_2_. It is interesting to note that under low and medium pressure, the activated carbon with -SO_3_H has the largest adsorption capacity, while, at high pressure, the activated carbon with -COOH shows the best adsorption performance. The reason is that, when the pressure is high, the adsorption capacity is determined by the pore volume, and the activated carbon with -COOH has a larger pore volume (see Table 1). Figure 2b plots the adsorption heat of the different activated carbons. It can be observed that the adsorption heat is dominated by the type of functional group. The average adsorption heat of the activated carbon with -COOH, -SO_3_H, -NH_2_, and -OH was 16.24 kcal/mol, 16.32 kcal/mol, 11.60 kcal/mol, and 12.80 kcal/mol, respectively.

**Figure 3:**
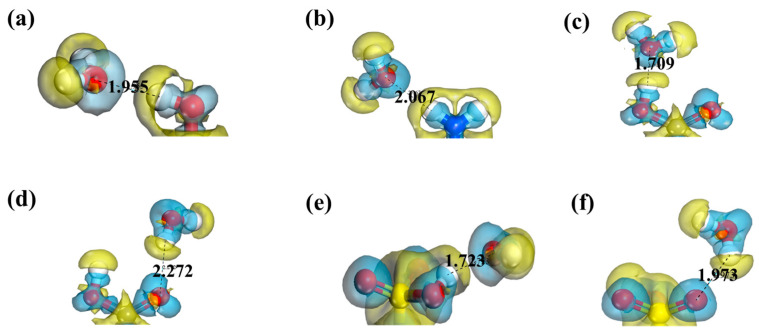
The cloud images of the charges of different functional groups and water molecules. (**a**) -OH, (**b**) -NH_2_, (**c**,**d**) -COOH, (**e**,**f**) -SO_3_H. Yellow represents atom losing its electron and carrying a certain positive charge, and blue represents atom attracting other electrons and carrying a certain negative charge.

The main reason for the high adsorption capacity of activated carbon with functional groups is the electrostatic force between functional groups and water molecules. As a result, the water molecules are adsorbed around functional groups in the form of hydrogen bonds. The formation process of hydrogen bonds between different functional groups and water is simulated by the CASTEP module of MS. Figure 3 presents the cloud images of charge and hydrogen bonds between water molecules and different functional groups. In these figures, yellow represents an atom losing its electron so that it carries a certain positive charge, and blue represents an atom attracting another electron so that it carries a certain negative charge.

It can be seen from Figure 3a that the hydrogen atom in -OH with a certain positive charge can attract the oxygen atoms in water molecules, forming a hydrogen bond with a bond length of ~1.955 Å. Figure 3b shows that the hydrogen atoms in -NH_2_ can form hydrogen bonds with the oxygen atom in water molecules with a bond length of ~2.067 Å. Since there are two hydrogen atoms in -NH_2_, one -NH_2_ functional group may, in theory, form two hydrogen bonds. Figure 3c,d demonstrate that the hydrogen atom and oxygen atom linked to the carbon atom by a carbon–oxygen double bond can form two different hydrogen bonds with bond lengths of ~1.709 Å and ~2.272 Å, respectively. The calculated results prove that three atoms in the -SO_3_H can form hydrogen bonds with water molecules, i.e., the two oxygen atoms linked with sulfur atoms by a double bond and a hydrogen atom. The hydrogen bond length of the former is approximately ~1.973 Å, and that of the latter is about ~1.723 Å.

In general, the shorter the hydrogen bond, the stronger the hydrogen bond. The functional groups with shorter hydrogen bonds have higher adsorption performance. For example, the -COOH has much better water adsorption performance than the -NH_2_. In addition, it is not hard to find that the adsorption capacity of activated carbon with functional groups depends not only on the strength of hydrogen bonds but also on the number of adsorption sites provided by a single functional group. For instance, although the hydrogen bond strength between -NH_2_ and water molecules is weaker than that between -OH and water molecules, the activated carbon with -NH_2_ has a larger adsorption capacity than carbon with -OH since -NH_2_ can provide two hydrogen atoms to form hydrogen bonds with water molecules. Accordingly, under low and medium pressure, the activated carbon with -SO_3_H has the best water adsorption performance because -SO_3_H can form three hydrogen bonds.

### 2.2. The Influence of Functional Group Numbers

**Figure 4:**
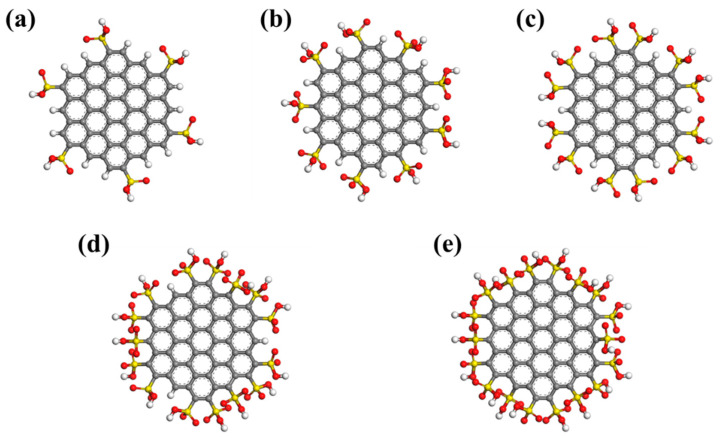
Graphene sheets with different numbers of -SO_3_H functional groups: (**a**) 6 -SO_3_H; (**b**) 9 -SO_3_H; (**c**) 12 -SO_3_H; (**d**) 15 -SO_3_H; (**e**)18 -SO_3_H. White atom represents H atom, red atom represents O atom, gray atom represents C atom, and yellow atom represents S atom.

In order to investigate the influence of the functional group number on the water adsorption capacity of activated carbon, different activated carbon models were built using 19-ring graphene sheets with 6, 9, 12, 15, and 18 -SO_3_H functional groups, respectively (see Figure 4).

**Figure 5:**
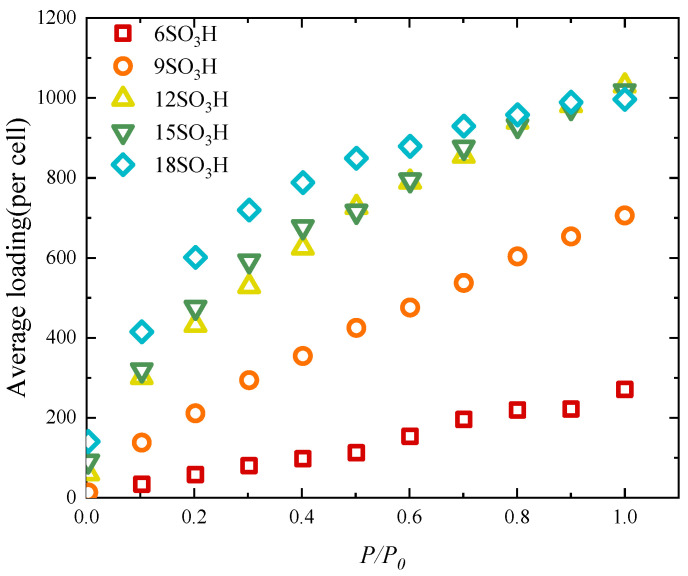
The adsorption isotherm of activated carbon with different numbers of -SO_3_H.

Figure 5 shows the adsorption isotherm of activated carbon when *T* = 298 K. It can be seen that, under low and medium pressure, the water adsorption increases as the number of functional groups increases. However, when the pressure is high and the number of functional groups is large, further increasing the functional groups may decrease water adsorption. When *P*/*P*_0_ = 1.0, the water adsorption amount of the activated carbon with 12 -SO_3_H is slightly larger than those of the activated carbons with 15 and 18 -SO_3_H. This is because, when the number of functional groups is large enough, as the functional group number increases, the total volume fraction decreases and there is less room for water molecules.

**Figure 6:**
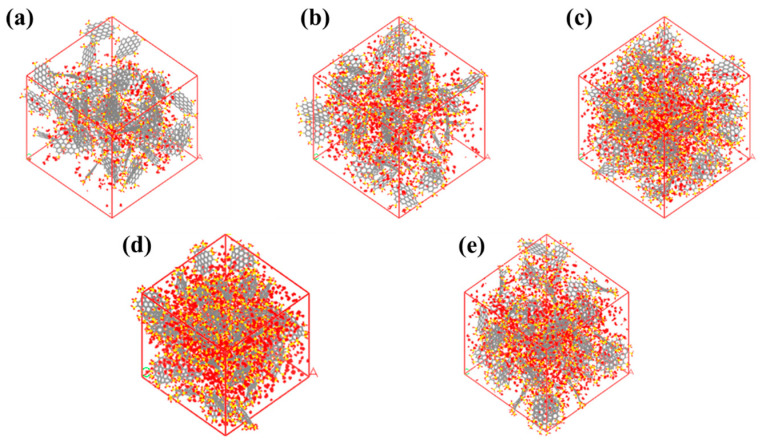
The adsorption sites of activated carbon with different numbers of -SO_3_H: (**a**) 6 -SO_3_H; (**b**) 9 -SO_3_H; (**c**) 12 -SO_3_H; (**d**) 15 -SO_3_H; (**e**) 18 -SO_3_H.

Figure 6 shows the adsorption sites for water on activated carbon when *T* = 298 K and *P*/*P*_0_ = 1.0. For the activated carbons with 6 -SO_3_H and 9 -SO_3_H, there are only a few adsorption sites, indicating that water molecules do not occupy the entire space. However, the adsorption sites are spread over the whole space in the activated carbon with 12 -SO_3_H. This means that, in this circumstance, the main factor determining the adsorption capacity is the pore volume fraction. Further increasing the numbers of functional groups would decrease the pore volume and thus reduce the adsorption capacity.

### 2.3. The Influence of Temperature

Temperature is one of the critical parameters for heat energy storage systems, which significantly influences the adsorption capacity of activated carbons. Thus, this section explores the influence of temperature on the water adsorption capacity and the adsorption heat of activated carbons in the temperature range of 323~473 K.

**Figure 7:**
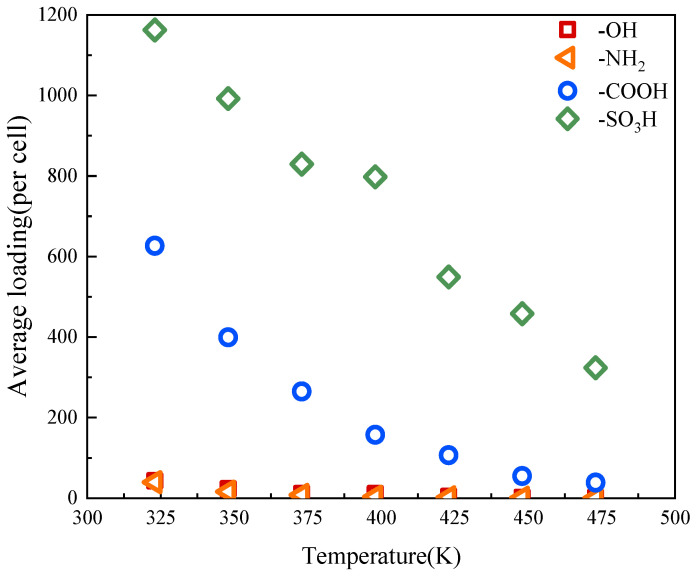
Average water adsorption of activated carbon with different functional groups, varying with temperature.

Figure 7 plots the average water adsorption of activated carbon with different functional groups as a function of temperature. As the temperature increases, the adsorption capacity of water decreases sharply. When the temperature increases from 323 K to 475 K, the average water adsorption amount of activated carbon with -SO_3_H decreases from 1162 per cell to 324 per cell, which is a reduction of 72%. Correspondingly, the water adsorption of activated carbon with -COOH decreases by 94%. When the temperature increases up to 473 K, the activated carbons with -NH_2_ and -OH almost lose their water adsorption capacities. This is because, as temperature increases, the hydrogen bonds between functional groups and water molecules break, and thus the water adsorption capacity of carbon decreases. In addition, since the hydrogen bonds between -NH_2_ and -OH are weak, they break more easily as the temperature increases.

**Figure 8:**
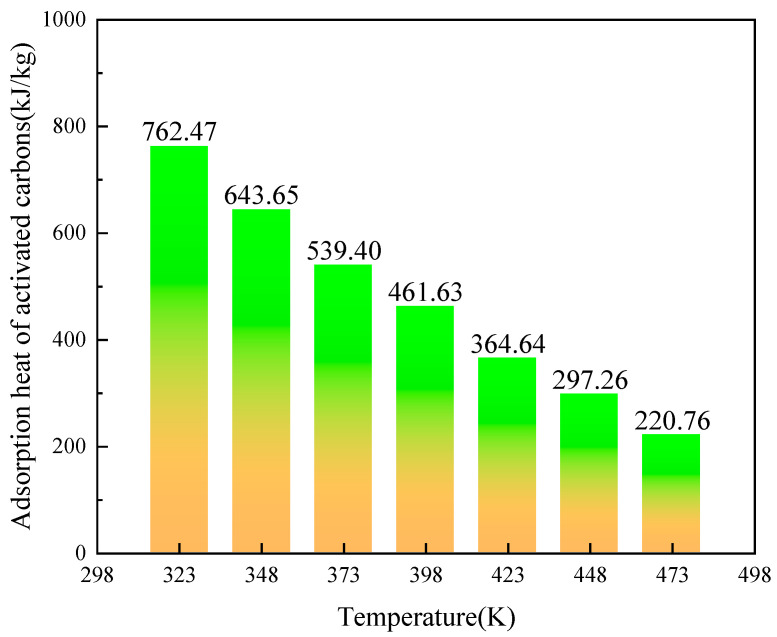
The adsorption heat per unit mass of activated carbon with -SO_3_H.

Figure 8 presents the adsorption heat per unit mass of activated carbon with different -SO_3_H, varying with temperature. It can be seen that, as temperature increases, the adsorption heat decreases. At the temperature of 323 K, the adsorption heat of the activated carbon can be up to 762.47 kJ/kg, which is much higher than the latent heat of phase change of traditional phase change materials [38]. Even at *T* = 473 K, the adsorption heat can be up to 220.76 kJ/kg, which is still higher than the latent heat of many phase change materials [39].

## 3. Physical Model and Numerical Details

### 3.1. Activated Carbon Model

**Figure 9:**
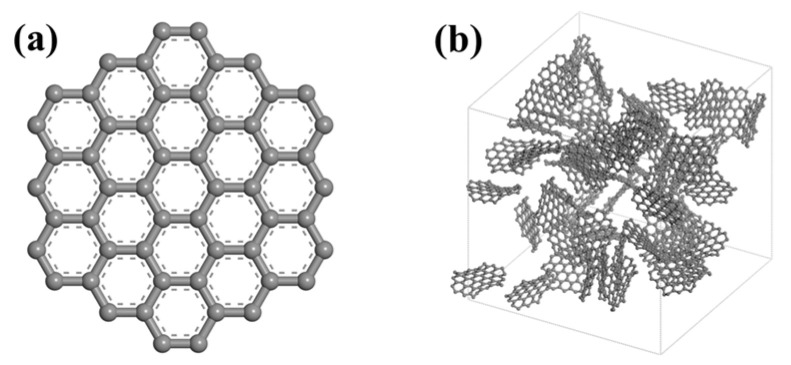
Graphene fragment and activated carbon model: (**a**) 19-ring graphene fragment; (**b**) disordered random model of activated carbon.

In order to precisely describe the actual pore structure of the activated carbon, scholars have come up with several different geometries (e.g., slit pore, random structure, and foam) [40,41,42]. Compared to other models, the random structure model can characterize the disorder and randomness of the activated carbon and is widely used. For instance, Gu et al. [43], Li et al. [44], and Su et al. [45] have researched the adsorption characteristics of organic volatiles in activated carbon with the help of a random structure model. Thus, this paper also employs the random structure model to describe activated carbon’s porous structure. The 19-ring graphene fragment, as shown in Figure 9a, is used as the basic unit. A disordered random model of activated carbon is established by randomly seeding a certain number of 19-ring graphene fragments into a three-dimensional cubic unit cell (50 Å × 50 Å × 50 Å), as shown in Figure 9b. Then, the activated carbon model with different functional groups is built by adding different types and numbers of functional groups to unsaturated carbon atoms with the Sketch Fragment program in MS.

**Figure 10:**
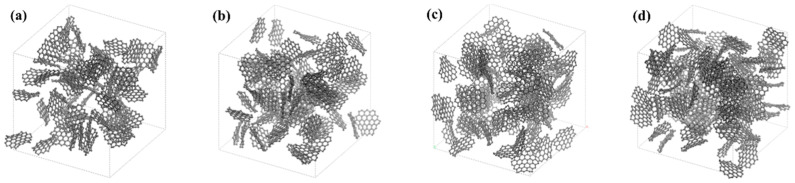
Activated carbon models with different densities: (**a**) 0.50 g/cm^3^; (**b**) 0.61 g/cm^3^; (**c**) 0.70 g/cm^3^; (**d**) 0.80 g/cm^3^.

**Figure 11:**
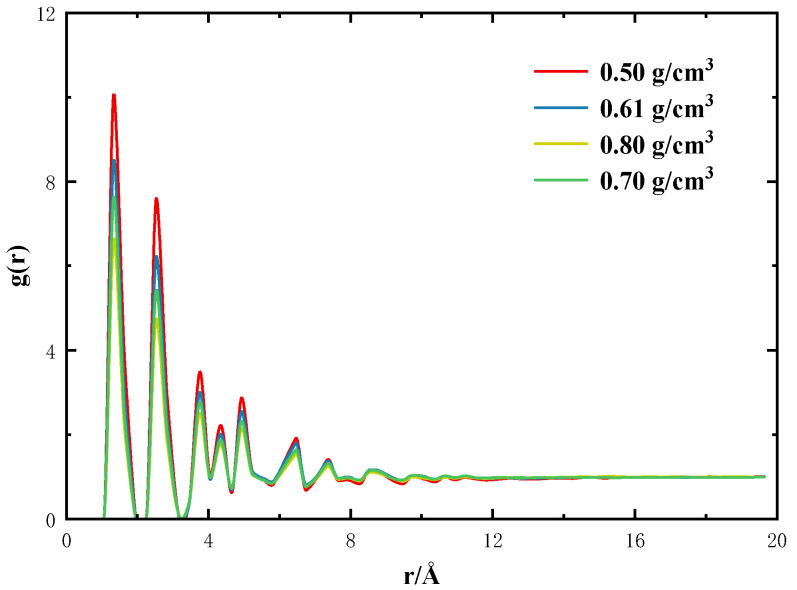
Radial distribution function of activated carbon with different densities.

The radial distribution function (RDF) can be physically expressed as the ratio of local density to bulk density, as follows:

$$g(r)=\frac{dN}{\rho 4\pi r^{2}dr}$$

where *dN* is the number of atoms in the area that form the reference atom “*r*” to “*r* + *dr*”. Theoretically, when the distance from the reference atom is farther away, the value of the radial distribution function (RDF) tends to 1. In order to judge the rationality of the spatial structure of the random model, we calculated the RDF of the activated carbon at different densities (0.50 g/cm^3^, 0.61 g/cm^3^, 0.70 g/cm^3^, 0.80 g/cm^3^) (Figure 10). As shown in Figure 11, the peak of the RDF of the activated carbon model at different densities appears at the same distance, and the RDF eventually converges to 1, which may prove the rationality of the structure of the activated carbon model constructed in this paper.

### 3.2. Theory and Calculation Details

In this work, we investigated the adsorption process and adsorption heat of water by active carbon by using Metropolis Monte Carlo in the SORPTION package. Choosing the appropriate force field to describe the forces acting between atoms is key to accurately simulating the adsorption process. The COMPASS force field is the first high-quality force field to integrate the parameters of organic and inorganic materials, and which can accurately and simultaneously predict the gas-phase properties (e.g., structure, conformation, and vibration) and condensed phase properties (e.g., equations of state and cohesion) of various molecules and polymers. In this force field, every atom is regarded as a nonbonded particle, and interaction energy between atoms is calculated using the following formula:

$$E_{\mathrm{pot}}=\sum_{i>j}\frac{q_{i}q_{j}}{r_{ij}}+\sum_{i>j}\varepsilon_{ij}[2{\left(\frac{r_{ij}^{0}}{r_{ij}}\right)}^{9}-3{\left(\frac{r_{ij}^{0}}{r_{ij}}\right)}^{6}]$$

where *ε_ij_* and *r_ij_*^0^ are the energy and length parameters of the Lennard–Jones 6–9 potential, respectively. Thus, we chose the COMPASS force field to simulate the adsorption process. Further, the force field, Ewald & Group, and Atom based were employed for calculating the atomic charge, electrostatic interaction, and van der Waals interaction energy, respectively. The temperature was set to 298 K, and the number of balancing steps and the number of data output steps were set to 10 × 10^7^ and 10 × 10^6^.

In addition, we also studied the electric density difference and hydrogen bond between functional groups on the surface of activated carbon and water using density functional theory. In this work, the Perdew–Burke–Ernzerhof (PBE) function was chosen to calculate the electric density difference and hydrogen bond between functional groups on the surface of activated carbon and water. It has been proven to be able to exactly describe the exchange–correlation interaction [46].

## 4. Conclusions

In the present work, we investigated the influence of the categories and numbers of functional groups on the water adsorption capacity of activated carbon using the GCMC method. The adsorption heat of activated carbon with different functional groups was also calculated and compared. In addition, the adsorption mechanism between functional groups and water molecules was analyzed using density functional theory. The main conclusions are drawn as follows:

1. The activated carbon without functional groups is superhydrophobic and hardly absorbs water under low and medium pressure. The functional groups can significantly improve the water adsorption capacity of activated carbon. Under low and medium pressure, the activated carbon with -SO_3_H shows the best adsorption capacity, followed by the activated carbon with -COOH. The functional groups of -OH and -NH_2_ can also improve the adsorption capacity of the activated carbon but are not as effective as -SO_3_H and -COOH.
2. Under low and medium pressure, increasing the number of -SO_3_H functional groups can increase the water adsorption capacity. However, increasing the functional group numbers may decrease the water adsorption capacity due to porosity reduction when the pressure is high. The length and number of hydrogen bonds between functional groups and water molecules affect the adsorption capacity significantly.
3. In the temperature range of 323~473 K, the adsorption capacity of activated carbons with -OH, -NH_2_, -COOH, and -SO_3_H decreases with temperature increase. The activated carbon with -SO_3_H exhibits excellent adsorption performance and the prospect of application for heat storage.

## Figures and Tables

**Table 1:** Structural parameters of different activated carbon models.

Functional Group	The Solid Volume, Å^3^	The Pore Volume, Å^3^	Porosity, %
--	33,305	91,695	73.36
-OH	42,230	82,770	66.22
-NH2	47,797	77,203	61.76
-COOH	68,992	56,008	44.81
-SO_3_H	84,344	40,656	32.52

**Table 2:** The charge of each atom in different activated carbon models.

	C	H	O	N	S
--	−0.1268	0.1268	/	/	/
-OH	0.042	0.41	−0.452	/	/
-NH_2_	0.01	0.353	/	−0.716	/
-COOH	0.53/−0.035	0.41	−0.45/−0.455	/	/
-SO_3_H	0.16	0.41	−0.543/−0.535	/	1.043

## Data Availability

Data are contained within the article.

## References

[1] BP (2020). Statistical Review of World Energy/Energy in 2020: The Year of COVID, (n.d.) 22.

[2] Zhang S., Li Z., Wang H., Tian L., Jin Y., Alston M., Yan Y. (2022). Component-dependent thermal properties of molten salt eutectics for solar thermal energy storage: Experiments, molecular simulation and applications. Appl. Therm. Eng..

[3] Costa S.C., Kenisarin M. (2022). A review of metallic materials for latent heat thermal energy storage: Thermophysical properties, applications, and challenges. Renew. Sustain. Energy Rev..

[4] Faraj K., Khaled M., Faraj J., Hachem F., Castelain C. (2021). A review on phase change materials for thermal energy storage in buildings: Heating and hybrid applications. J. Energy Storage.

[5] Lefebvre D., Tezel F.H. (2017). A review of energy storage technologies with a focus on adsorption thermal energy storage processes for heating applications. Renew. Sustain. Energy Rev..

[6] Di Palo M., Sabatelli V., Buzzi F., Gabbrielli R. (2020). Experimental and Numerical Assessment of a Novel All-In-One Adsorption Thermal Storage with Zeolite for Thermal Solar Applications. Appl. Sci..

[7] Gautam A., Saini R.P. (2020). A review on technical, applications and economic aspect of packed bed solar thermal energy storage system. J. Energy Storage.

[8] Nasruddin, Arsyad A.P., Djubaedah, Wulandari D.A., Hidayat H.F. (2020). Experimental analysis of Indonesian natural zeolites-water pair for closed system adsorption thermal energy storage. AIP Conf. Proc..

[9] Miller M.A., Wang C.-Y., Merrill G.N. (2009). Experimental and Theoretical Investigation Into Hydrogen Storage via Spillover in IRMOF-8. J. Phys. Chem. C.

[10] Kohler T., Müller K. (2017). Storage of low grade solar thermal energy by adsorption of organics. AIP Conf. Proc..

[11] Hu Q., Lu Y., Meisner G.P. (2008). Preparation of Nanoporous Carbon Particles and Their Cryogenic Hydrogen Storage Capacities. J. Phys. Chem. C.

[12] Deshmukh H., Maiya M.P., Srinivasa Murthy S. (2017). Study of sorption based energy storage system with silica gel for heating application. Appl. Therm. Eng..

[13] Ayisi E.N., Fraňa K. (2020). The Design and Test for Degradation of Energy Density of a Silica Gel-Based Energy Storage System Using Low Grade Heat for Desorption Phase. Energies.

[14] Shi W., Zhu Y., Shen C., Shi J., Xu G., Xiao X., Cao R. (2019). Water sorption properties of functionalized MIL-101(Cr)-X (X = –NH_2_, –SO_3_H, H, –CH_3_, –F) based composites as thermochemical heat storage materials. Microporous Mesoporous Mater..

[15] Srinivasu K., Ghosh S.K. (2011). Tuning the Metal Binding Energy and Hydrogen Storage in Alkali Metal Decorated MOF-5 through Boron Doping: A Theoretical Investigation. J. Phys. Chem. C.

[16] Liang Q., Liu Y., Chen M., Ma L., Yang B., Li L., Liu Q. (2020). Optimized preparation of activated carbon from coconut shell and municipal sludge. Mater. Chem. Phys..

[17] Sultan M., El-Sharkaw I.I., Miyazaki T., Saha B.B., Koyama S. (2014). Experimental Study on Carbon Based Adsorbents for Greenhouse Dehumidification. Evergreen.

[18] Chairunnisa, Mikšík F., Miyazaki T., Thu K., Miyawaki J., Nakabayashi K., Wijayanta A.T., Rahmawati F. (2020). Enhancing water adsorption capacity of acorn nutshell based activated carbon for adsorption thermal energy storage application. Energy Rep..

[19] Wiig E.O., Juhola A.J. (1949). The Adsorption of Water Vapor on Activated Charcoal. J. Am. Chem. Soc..

[20] Qian Q., Sunohara S., Kato Y., Zaini M.A.A., Machida M., Tatsumoto H. (2008). Water vapor adsorption onto activated carbons prepared from cattle manure compost (CMC). Appl. Surf. Sci..

[21] Horikawa T., Sakao N., Do D.D. (2013). Effects of temperature on water adsorption on controlled microporous and mesoporous carbonaceous solids. Carbon.

[22] Fletcher A.J., Uygur Y., Thomas K.M. (2007). Role of Surface Functional Groups in the Adsorption Kinetics of Water Vapor on Microporous Activated Carbons. J. Phys. Chem. C.

[23] Tangsathitkulchai C., Ngernyen Y., Tangsathitkulchai M. (2009). Surface modification and adsorption of eucalyptus wood-based activated carbons: Effects of oxidation treatment, carbon porous structure and activation method. Korean J. Chem. Eng.

[24] Koyanagi J., Itano N., Yamamoto M., Mori K., Ishida Y., Bazhirov T. (2019). Evaluation of the mechanical properties of carbon fiber/polymer resin interfaces by molecular simulation. Adv. Compos. Mater..

[25] Xie J., Xiao C., Zhang L., Lü F., Xie Q., Cheng L. (2021). Molecular simulation of nano polyhedral oligomeric silsesquioxane doping effect on the properties of two-component crosslinked epoxy resin. J. Mol. Graph. Model..

[26] Muller M.P., Jiang T., Sun C., Lihan M., Pant S., Mahinthichaichan P., Trifan A., Tajkhorshid E. (2019). Characterization of Lipid–Protein Interactions and Lipid-Mediated Modulation of Membrane Protein Function through Molecular Simulation. Chem. Rev.

[27] Jiang Z., Zhang H., Bian X., Li J., Li J., Zhang H. (2019). Insight into the binding of ACE-inhibitory peptides to angiotensin-converting enzyme: A molecular simulation. Mol. Simul..

[28] Narayanaswamy V., Alaabed S., AL-Akhras M.-A., Obaidat I.M. (2020). Molecular simulation of adsorption of methylene blue and rhodamine B on graphene and graphene oxide for water purification. Mater. Today Proc..

[29] Chu X., Liu S., Zhou S., Zhao Y., Xing W., Lee C.-H. (2016). Adsorption behaviors of CO_2_ and CH_4_ on zeolites JSR and NanJSR using the GCMC simulations. Adsorption.

[30] Smykowski D., Szyja B., Szczygieł J. (2014). GCMC simulations of CO_2_ adsorption on zeolite-supported Ir4 clusters. J. Mol. Graph. Model..

[31] Fu H., Wang Y., Zhang T., Yang C., Shan H. (2017). Adsorption and Separation Mechanism of Thiophene/Benzene in MFI Zeolite: A GCMC Study. J. Phys. Chem. C.

[32] Chen L., Morrison C.A., Düren T. (2012). Improving Predictions of Gas Adsorption in Metal–Organic Frameworks with Coordinatively Unsaturated Metal Sites: Model Potentials, ab initio Parameterization, and GCMC Simulations. J. Phys. Chem. C.

[33] Peng X., Cheng X., Cao D. (2011). Computer simulations for the adsorption and separation of CO_2_/CH_4_/H_2_/N_2_ gases by UMCM-1 and UMCM-2 metal organic frameworks. J. Mater. Chem.

[34] Camp J., Stavila V., Allendorf M.D., Prendergast D., Haranczyk M. (2018). Critical Factors in Computational Characterization of Hydrogen Storage in Metal–Organic Frameworks. J. Phys. Chem. C.

[35] Li X., Xue Q., He D., Zhu L., Du Y., Xing W., Zhang T. (2017). Sulfur–Nitrogen Codoped Graphite Slit-Pore for Enhancing Selective Carbon Dioxide Adsorption: Insights from Molecular Simulations. ACS Sustain. Chem. Eng..

[36] Wang S., Lu L., Wu D., Lu X., Cao W., Yang T., Zhu Y. (2016). Molecular Simulation Study of the Adsorption and Diffusion of a Mixture of CO_2_/CH_4_ in Activated Carbon: Effect of Textural Properties and Surface Chemistry. J. Chem. Eng. Data.

[37] Huang L., Zhang L., Shao Q., Lu L., Lu X., Jiang S., Shen W. (2007). Simulations of Binary Mixture Adsorption of Carbon Dioxide and Methane in Carbon Nanotubes: Temperature, Pressure, and Pore Size Effects. J. Phys. Chem. C.

[38] Rathod M.K., Banerjee J. (2013). Thermal stability of phase change materials used in latent heat energy storage systems: A review. Renew. Sustain. Energy Rev..

[39] Sutjahja I.M., Rahayu A.U.S., Kurniati N., Pallitine I.D., Kurnia D. (2016). The role of chemical additives to the phase change process of CaCl_2_ 6H2O to optimize its performance as latent heat energy storage system. J. Phys. Conf. Ser..

[40] Striolo A., Chialvo A.A., Cummings P.T., Gubbins K.E. (2003). Water Adsorption in Carbon-Slit Nanopores. Langmuir.

[41] Kumar K.V., Salih A., Lu L., Müller E.A., Rodríguez-Reinoso F. (2011). Molecular Simulation of Hydrogen Physisorption and Chemisorption in Nanoporous Carbon Structures. Adsorpt. Sci. Technol..

[42] Sihn S., Roy A.K. (2004). Modeling and prediction of bulk properties of open-cell carbon foam. J. Mech. Phys. Solids.

[43] Gu Z.-K., Zhu C.-Y., Huang Z.-Q., Xu M.-H., Gong L. (2021). Numerical simulations on the adsorption characteristics of aromatics on activated carbon by the GCMC method. Case Stud. Therm. Eng..

[44] Li S., Song K., Zhao D., Rugarabamu J.R., Diao R., Gu Y. (2020). Molecular simulation of benzene adsorption on different activated carbon under different temperatures. Microporous Mesoporous Mater..

[45] Su Z., Zhang Y., Huang L., Wang S., Zhu Y., Li L., Lu X. (2020). Acetone adsorption on activated carbons: Roles of functional groups and humidity. Fluid Phase Equilibria.

[46] Ao Z.M., Li S., Jiang Q. (2010). Correlation of the applied electrical field and CO adsorption/desorption behavior on Al-doped graphene. Solid State Commun..

